# Weight Loss Maintenance in African American Women: A Systematic Review of the Behavioral Lifestyle Intervention Literature

**DOI:** 10.1155/2013/437369

**Published:** 2013-04-11

**Authors:** Lisa M. Tussing-Humphreys, Marian L. Fitzgibbon, Angela Kong, Angela Odoms-Young

**Affiliations:** ^1^Department of Medicine, University of Illinois at Chicago, Chicago, IL 60608, USA; ^2^School of Public Health, University of Illinois at Chicago, Chicago, IL 60608, USA; ^3^Cancer Education and Career Development Program, Institute of Health Research and Policy, University of Illinois at Chicago, Chicago, IL 60608, USA; ^4^Department of Kinesiology and Nutrition, University of Illinois at Chicago, Chicago, IL 60608, USA

## Abstract

We performed a systematic review of the behavioral lifestyle intervention trials conducted in the United States published between 1990 and 2011 that included a maintenance phase of at least six months, to identify intervention features that promote weight loss maintenance in African American women. Seventeen studies met the inclusion criteria. Generally, African American women lost less weight during the intensive weight loss phase and maintained a lower % of their weight loss compared to Caucasian women. The majority of studies failed to describe the specific strategies used in the delivery of the maintenance intervention, adherence to those strategies, and did not incorporate a maintenance phase process evaluation making it difficult to identify intervention characteristics associated with better weight loss maintenance. However, the inclusion of cultural adaptations, particularly in studies with a mixed ethnicity/race sample, resulted in less % weight regain for African American women. Studies with a formal maintenance intervention and weight management as the primary intervention focus reported more positive weight maintenance outcomes for African American women. Nonetheless, our results present both the difficulty in weight loss and maintenance experienced by African American women in behavioral lifestyle interventions.

## 1. Introduction

Overweight (body mass index (BMI) 25.0–29.9 kg/m^2^) and obesity (BMI ≥ 30 kg/m^2^) are global public health problems [1, 2]. All demographic sectors of the United States (US) population are affected, but African American (AA) women are disproportionately burdened [1, 3]. As reported in 2012 (National Health and Nutrition Examination Survey (NHANES), 2009-2010), approximately 82% of AA adult women in the US were classified as overweight or obese [1]. This disparity is of particular concern given that overweight and obesity are associated with a number of serious chronic diseases [4, 5]. 

The most common approach to obesity treatment includes lifestyle interventions that target both diet and physical activity (PA) and some form of behavioral self-management [6–9]. Traditionally, AA women enrolled in behavioral lifestyle interventions lose less weight when compared to other subgroups [3, 10–16] although even modest weight reduction improves the cardiovascular risk profile [17, 18] and decreases diabetes incidence [19]. If weight loss is not sustained, the health benefits of weight reduction are attenuated [20, 21]. This fact highlights the importance of understanding factors that support long-term weight control across populations. 

The challenge of maintaining weight loss is well documented [9, 22–24]. Typically, individuals regain about 30–35% of their initial weight loss within the first year following treatment, and more than half return to their baseline weight within five years [7, 25]. Data from NHANES (1999–2006) found that only 1 of 6 of the overweight/obese participants surveyed reported that they had ever maintained a weight loss of at least 10% for one year [26]. Contributors to weight regain include physiologic adaptations such as reduced resting energy expenditure [27] and leptin concentrations [28, 29], increased ghrelin (a gut peptide associated with hunger) [30, 31], and exposure to an obesogenic environment [32, 33]. Weight regain may also be related to the distinct differences between weight loss and weight loss maintenance behaviors [7, 22]. For example, during weight loss, foods that lead to weight gain are avoided, whereas with weight maintenance, food needs to be better managed, overall [7]. Despite these challenges, some individuals are successful at long-term weight maintenance. Behaviors associated with successful weight loss maintenance identified through the use of the National Weight Control Registry, which consists of more than 6000 adults who have lost at least 13.6 kg and maintained this loss for a minimum of one year, report that successful weight loss maintainers consume a low-fat diet, eat breakfast, weigh themselves regularly, and report high levels of both dietary restraint and PA [34, 35]. Although these data are encouraging, the Registry is comprised predominately of Caucasian women, making it a less representative sample. 

Minorities, including AA women, are largely underrepresented in the behavioral lifestyle intervention literature, however, two systematic reviews addressing obesity treatment in minority populations were recently published [36, 37]. Reviews concluded that cultural adaptations [38], church-based studies [39], a low carbohydrate diet plan [40], individual sessions [38], family-centered programs [41, 42], and problem-solving skills [43, 44] promoted both weight loss and maintenance in minority adults. A third review, focusing specifically on AA women [45], concluded that attention to cultural preferences, behavioral management strategies, and session attendance were important factors to successful weight loss. However, to the best of our knowledge, no studies have examined the existing behavioral lifestyle intervention literature to identify potential strategies that are effective in promoting long-term weight control specific to AA women. Therefore, our objective was to systematically review, synthesize, and summarize the behavioral lifestyle intervention literature to evaluate the effectiveness of these interventions on weight loss maintenance in AA women. These results can then better inform the design of future weight management interventions for this population. 

*Note*. We recognize that the racial/ethnic category “African American” describes a diverse group of people descended from many different cultures of Africa and the Caribbean including those whose families have lived in the US for centuries and those who more recently emigrated. We will use this term to broadly characterize the individuals discussed in this manuscript. 

## 2. Methods

The systematic review focused on the behavioral lifestyle intervention literature published between 1990 and 2011. The year 1990 was chosen as a starting point because “Healthy People 2000,” which was the first comprehensive preventive health agenda for the US population, established specific goals for reducing the prevalence of overweight and obesity [46]. The authors referred to the guidelines recommended by the Preferred Reporting Items for Systematic Reviews and Meta-Analyses (PRISMA) checklist for collection, synthesis, and reporting of the data for the systematic review [47]. References were identified through a search of MEDLINE via PubMed, CINAHL Plus, and Academic Search Premier databases. The authors defined weight loss maintenance as period of at least six months, with or without inclusion of a formal maintenance program, following participation in an intensive behavioral lifestyle intervention in which weight was an outcome. Search terms included a combination of the following: *weight loss maintenance, long-term weight loss, weight regain, weight loss, dietary intervention, obesity, AA, and black*. We also used the “ancestry approach” [48] by reviewing the reference sections of pertinent papers as well as past review articles focused on weight loss maintenance. 

Randomized and nonrandomized studies were included in the review if they met the following criteria: (1) English language papers published in peer-reviewed journals, (2) behavioral lifestyle interventions with a maintenance phase of at least six months (both formal maintenance programs and non-contact periods) in which weight was reported as an outcome, (3) studies conducted in the US (due to potential country-specific differences in weight management practices) [49], (4) adult participants at least 18 years of age, (5) inclusion of AA women, and (6) weight outcomes reported separately by ethnicity/race and sex. Studies were excluded if they: (1) were published in a language other than English, (2) excluded AA women, (3) had a maintenance period less than six months (both formal maintenance programs and non-contact periods), (4) included pregnant or postpartum women, (5) primarily focused on a surgical or pharmacological weight loss intervention, (6) provided prepared meals, (7) omitted weight outcomes for the maintenance phase, (8) were not an intervention study, or (9) included a pediatric sample. Studies that used liquid meal replacements as the primary intervention were also excluded, although studies in which meal replacements were used as one component of an intervention were included. Finally, due to the paucity of studies reporting on this topic, authors of eligible studies that did not report results by race/ethnicity and/or sex were contacted by email to inquire if such information could be provided. Weight-related data by race/ethnicity and sex were obtained through this method for four studies [3, 16, 44, 50] but could not be gleaned from the primary authors for four other interventions and were thus excluded [51–54].

**Figure 1:**
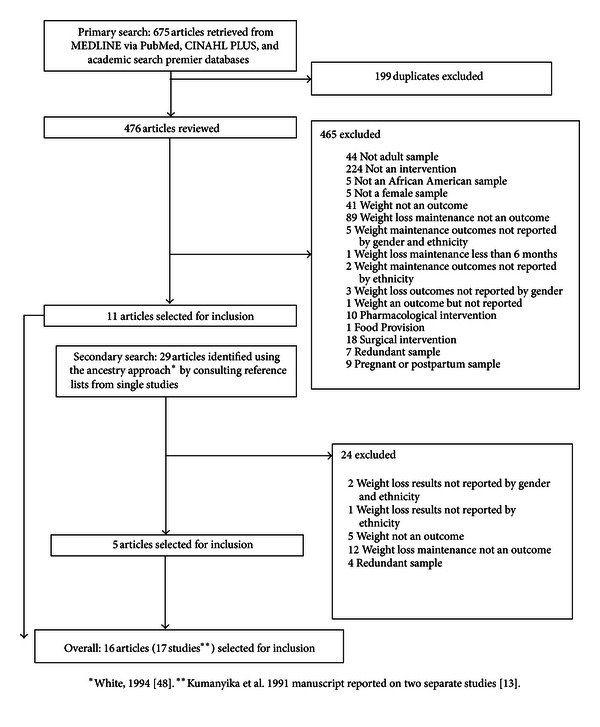
Article search results.

Figure 1 presents the study attrition diagram and the number of publications included at each step during the search process. The initial search, utilizing the three databases, yielded 675 publications. After eliminating duplicates, the total was reduced to 476 papers. The authors L. M. Tussing-Humphreys, A. Kong, and M. L. Fitzgibbon completed an initial screening using article abstracts or full articles, where necessary, to determine eligibility. To avoid bias, the studies in which M. L. Fitzgibbon was primary or coauthor, L. M. Tussing-Humphreys and A. Kong reviewed the abstracts and articles for study inclusion. After reviewing the abstracts and/or full text from the primary search, 465 articles were excluded, leaving 11 papers. The secondary search, using the “ancestry approach” [48], resulted in the identification of 28 additional articles. The abstracts for these articles were reviewed as described previously and resulted in the inclusion of 5 additional articles. In total, 16 papers met our inclusion criteria. However, one article [13] reported weight loss results for two separate multicenter hypertension trials (Hypertension Prevention Trial and the Trials of Hypertension Prevention) and thus was coded as two separate studies, resulting in a total of 17 trials.

For each of the 17 studies, the primary author (L. M. Tussing-Humphreys) extracted the following data, using a standardized form, which are presented in Tables 1 and 2: (1) author and year of publication; (2) study design, setting, and duration of the trial; (3) participant characteristics including sample size, age, income, education, and health status; (4) overarching intervention characteristics including use of a formal theoretical framework inclusion of cultural adaptations, duration of the maintenance phase, *a priori* criteria for entry into the maintenance period, and components targeted at weight loss maintenance; (5) frequency, format, and dose of maintenance intervention sessions or contacts; (6) mean baseline weight in kg; (7) change in weight (kg) immediately following the active intervention phase; (8) weight change in kg from baseline; (9) % weight regain at several reported follow-up intervals (12 months, 18 months, and end of trial when available) (several time points were selected in an attempt to compare weight changes during the maintenance phase across studies); and (10) adherence to maintenance sessions or components and study retention (defined as % of participants available at designated postintervention follow-up time-point). Where possible, missing variables were calculated or estimated from data reported or from figures presented in the paper or obtained directly from the study authors. Percent weight regain was crudely calculated for all studies using available weight change data. For trials in which multiple articles were published (e.g., Diabetes Prevention Program (DPP), Weight Loss Maintenance Trial (WLM), the Hypertension Prevention Trial (HPT), the Trial of Hypertension Prevention (TOHP), the Trial of Hypertension Prevention II (TOHP II), the Trial of Nonpharmacologic Interventions in the Elderly (TONE), and The Treatment of Obesity in Underserved Settings (TOURS)), we incorporated all relevant data regardless if the source was other than the study reporting weight loss by race/ethnicity and sex. Data extracted from the 17 studies were reviewed by two of the coauthors for accuracy (M. L. Fitzgibbon and A. Kong). 

To address study quality, we adapted the ranking system developed by Whitt-Glover and Kumanyika [71] which was designed to evaluate both randomized and nonrandomized studies. Nonrandomized studies were included to allow for insight regarding potentially effective strategies utilized in studies with a less rigorous design and due to the paucity of literature published on the topic. The study quality ranking criteria are described herein after.

*Study Design.* The ranking system was 1 for uncontrolled studies, 2 for nonrandomized controlled studies, 3 for randomized controlled pilot studies, and 4 for full-scale randomized controlled trials (RCTs). Full-scale RCTs were deemed the highest-quality study design because (1) random assignment to treatment tends to minimize selection bias, (2) treatment and control groups are similar in characteristics and sample size, and (3) equality of treatment arms produces valid statistical tests [72].

*Degree of Focus on Weight Control.* The ranking system was 1 for studies in which weight control was not a primary focus of the intervention and 2 for studies in which weight control was the primary focus. Our working assumption was that studies in which the intervention content was focused on weight control would produce better weight change and maintenance outcomes than studies in which weight control was not the primary focus. 

*Inclusion of Formal Weight Maintenance Intervention.* The ranking system was 1 for interventions with no formal maintenance intervention and 2 for studies that included a formalized maintenance phase. The assumption was that interventions that included formal maintenance treatment would produce better long-term weight control for participants compared to interventions with minimal or no contact during the maintenance period. Extended care following a period of intensive behavioral treatment has shown to be effective in producing long-term weight control [73]. 

*Cultural Adaptations.* The ranking system was 1 for studies in which no cultural adaptations were reported, 2 for studies in which the only adaptation was limiting recruitment to AAs, and 3 for studies reporting attempts at adapting intervention-related content and other adaptations including staff trainings and oversight committees [71]. The working assumption was that cultural adaptations could affect acceptability, effectiveness, and retention. 

## 3. Results

### 3.1. Overall

The 17 studies are ranked alphabetically according to date published and study quality which ranged between 5 and a maximum of 11 points (Tables 1 and 2). Weight was the primary outcome for the majority of studies (15 of 17) [3, 11, 13–16, 44, 50, 56, 60–62, 65, 69]. However, one trial focused on increasing PA and improving dietary quality [67] and another focused on increasing daily steps [70]. Both trials reported weight outcomes, as a secondary endpoint, and were thus included in the review. 

### 3.2. Design, Setting, and Length of Intervention

Thirteen of the 17 studies were RCTs. The interventions were implemented in various settings including academic medical centers [3, 11, 13, 44, 50, 56, 65], five of which were multi-institution collaboratives [11, 13, 44, 56, 65], universities [14, 60, 62], medical clinics [16, 61, 69], and community-based locales [15, 67]. One study did not report the intervention setting [70]. The duration of the trials ranged from 12 to 36 months. 

### 3.3. Sample Size and Participant Characteristics

The sample sizes varied significantly across the studies ranging from 21 to 2921 participants. The multi-institution RCTs [11, 13, 44, 56, 65] and the pilot RCT by Tsai et al. [16] recruited participants of mixed race/ethnicity and sex. Notably, the weight loss treatment arms of the HPT [13], TOHP [13], and TONE [44] trials included relatively small numbers of AA women ranging from just 28 to 46 women. Six studies [60–62, 67, 69, 70] targeted recruitment specifically at AA women with sample sizes ranging from 21 to 366 women. Two studies recruited both AA and Caucasian women [14, 15], and two recruited AA men and women only [3, 50].

The majority of the studies (16 of 17) enrolled AA women with mean ages between 40 to 60 years old. All of the studies recruited overweight and obese individuals although their health status varied. Participants in the TONE trial [44] and study by Banks-Wallace [70] were hypertensive, participants in the WLM trial [56] were hypertensive and/or dyslipidemic, the DPP trial [11] participants presented with impaired glucose tolerance, West et al. [14] and McNabb et al. [69] recruited type 2 diabetics, and Djuric et al. [62] recruited breast cancer survivors. 

### 3.4. Overarching Intervention Characteristics: Use of Theoretical Framework and Cultural Adaptations

Eight studies utilized a formal theoretical framework in the design of the intervention. [3, 11, 44, 56, 60, 67, 74, 75]. Twelve studies [3, 11, 15, 44, 50, 56, 60–62, 67, 69] reported incorporating some form of cultural adaptation salient to AAs including recruitment of only AA participants [60–62, 67], culturally specific diet and PA modifications [3, 11, 50, 56, 60, 61, 67], cultural sensitivity training for research staff [44, 56], employing AA case managers and interventionists [3, 11, 50, 60, 67], special attention to religion and spirituality [60, 62], AA community-focused field-trips to grocery stores, parks, and so forth [50], selection of study site in an AA community [67], and the formation of a minority implementation committee [56].

### 3.5. Weight Loss Outcomes following the Intensive Intervention Phase

Across the 17 studies, weight changes for AA women following the intensive intervention phase ranged from +0.5 to −8.5 kg. In the studies enrolling both AA and Caucasian women [11, 13, 14, 16, 44, 56], initial weight loss for AA women ranged from −1.9 to −7.1 kg versus −3.4 to −10.7 kg for Caucasian women. The weight loss plus sodium reduction arm of the TONE trial [44] was the only treatment arm across the 17 studies in which initial weight losses were similar between AA (3.9 ± 3.6 kg) and Caucasian (3.9 ± 3.9 kg) women. 

### 3.6. Maintenance Phase Characteristics

The duration of the maintenance phase ranged from 6 to 30 months. Only, two studies [3, 56] reported inclusion criteria for entry into the maintenance phase. For the WLM trial [56], participants were required to have lost a minimum of 4 kg during the six-month active weight loss phase to be randomized to a maintenance treatment group. For the HELP study [3] participants were required to attend the postphase 1 data collection to be randomized to the maintenance phase. 

Common features of the maintenance interventions included some combination of didactic nutrition and PA sessions [3, 11, 13–15, 44, 50, 56, 60, 62, 65], promotion of adherence to the prescribed eating pattern or dietary modifications (e.g., calorie control, fat control, increased consumption of fruits, vegetables, and fiber) [3, 11, 13–15, 44, 50, 56, 60, 62, 65], achieving a set amount of PA (minutes or steps per day or week) [3, 11, 13–15, 44, 50, 56, 60, 62, 65], and ongoing emphasis on behavioral modification strategies learned during the active intervention phase including self-monitoring of weight, dietary intake, and PA, goal-setting, problem solving, relapse prevention, and stimulus control [3, 11, 13–15, 44, 50, 56, 60, 62, 65]. Importantly, the extent to which these topics were reviewed was difficult to discern from the manuscripts, as the needs of the participants often dictate what content is featured during the maintenance sessions. Additionally, supervised PA sessions were offered in three of the trials [11, 50, 60], and a number of more unique maintenance components were also tested including use of an individualized tool box [11, 76], internet-based delivery [60], motivational interviewing [14, 61], spirituality counseling [69], and family and friend support [15]. 

The frequency of contact and delivery of the maintenance interventions was diverse. Participants in seven studies [3, 16, 56, 61, 67, 69, 70] received no or minimal contact during the maintenance period. Six studies delivered the maintenance intervention through a combination of group and individual in-person or phone-based sessions [13, 14, 44, 50, 60, 65] with frequency ranging from twice weekly [60] to bimonthly [13]. Three studies conducted individual in-person or phone-based maintenance sessions [11, 15, 62]. Contact was made monthly in the personal contact arm of the WLM trial [56], at least monthly or as often as requested by participants in the DPP [11], and tapered from weekly, to biweekly, to monthly in the Djuric et al. trial [62]. The group maintenance arm of the HELP study [3] met solely in group sessions biweekly, during months 7–9, and lessened to monthly thereafter. Additionally, several studies mailed newsletters to participants at various times throughout the maintenance phase [13, 15, 50, 60, 62]. For the studies reporting dose of the maintenance sessions, encounters lasted anywhere from 2 to 90 minutes.

### 3.7. Adherence to Maintenance Sessions and Components

Participants, enrolled in four of the multi-institution RCTs, reported modest to excellent adherence to maintenance sessions [11, 13, 56, 65]. Unfortunately, adherence was not reported separately for AA women, and three of the multi-institution RCTs failed to report adherence to maintenance sessions altogether [13, 44]. In four studies, which included a formal maintenance intervention in which only AA adults were enrolled, adherence to maintenance sessions was paltry [3, 50, 60, 62]. In one of the single-site RCTs, with a mixed race/ethnicity sample, women attended less than 60% of the offered sessions [14], and in another [15], total counseling contact time, for the extended care maintenance treatment groups, exceeded the a *priori* estimate of 8.7 hours; results were not reported separately for AA women. 

The majority of studies, with a formal maintenance intervention, did not report adherence to specific maintenance activities such as self-monitoring of weight, dietary intake, or PA [3, 11, 13, 44, 50, 60, 62, 65]. One study [14] reported the mean number of food and activity diaries submitted throughout the intervention with submissions dropping from a mean of 15 (SD ± 8) diaries during the intensive phase of the program to only 5 (SD ± 9) diaries at 18-month follow-up. Rickel et al. [15] reported approximately 16 hours of journaling time for the extended care maintenance groups compared to just 10 hours in the self-directed group. The WLM trial reported that self-weighing was more frequent for AA women compared to Caucasian women [56]. However, like with session attendance, most studies with mixed race/ethnicity and/or sex sample [3, 11, 13–15, 50, 56] failed to report adherence to maintenance components altogether or separately for AA women making it difficult to discern any disparities. 

### 3.8. Study Retention

The percentage of participants available for final assessment varied. Five of the eight studies [11, 13, 44, 65] with a mixed race/ethnicity and/or sex sample reported retention rates separately for AA women. Retention rates for women randomized to an active intervention arm ranged from 48 to 97% for AA versus from 66 to 100% for Caucasian. Notably, retention rates were similar for the HPT [13], TOHP [13], and TOHPII [65] studies while retention was lower for AA women in the TONE [44] and DPP [11] trials. In the six trials enrolling only AA women [60, 61, 67, 69, 70] retention across treatment groups ranged from 63 to 92%. For the studies enrolling AA men and women [3, 50], retention for AA women ranged from 55% to 66% across treatments.

### 3.9. Weight Maintenance Outcomes and Maintenance Phase Characteristics

Table 2 reports the ratings for each quality category, a summary quality score, maintenance intervention characteristics, and % weight regain at 12, 18, and >18 months (calculated using available data). By summarizing the findings in this manner, we could more easily compare across interventions and determine if a particular set of maintenance intervention characteristics were more effective at promoting weight control for AA women. However, it is important to highlight the difficulty in comparing across studies given the heterogeneity in sample sizes, differences in duration of the maintenance phase, attrition rates, time interval in which weight outcomes were reported, and analysis approach (intention to treat versus completers); therefore, findings should be interpreted with some caution. With this acknowledgement, the 18 month weight outcomes were reported by a majority of the studies (14 of 17) [3, 11, 13–15, 44, 50, 60–62, 65, 67, 70] and will be used to make comparisons. 

At 18-month follow-up, % weight regain for AA women in studies with the highest quality ranking (11 points), enrolling only AA women [60] or AA adults [3, 50], ranged from 0 to 49%. In studies with a lower quality ranking (10 or less), % weight regain at 18-months ranged from 15 to 138%. Generally, the studies not focused on weight as an outcome [67, 70] or lacking a formalized maintenance program [61, 67, 70] had the poorest outcomes. 

The highest ranking studies (11 points), enrolling both AA and Caucasian women [11, 44], reported 18-month % weight regain ranging from 0 to 17% for AA women and from 12 to 17% for Caucasian women. Notably, in the TONE study [44], % weight regain was lower for AA women in both weight treatment arms throughout the maintenance period and % weight regain was only slightly higher for AA compared to Caucasian women in the DPP trial [11]. In the lower ranking studies (10 points or less) [13–15, 65], 18-month % weight regain for AA women ranged from 19 to 89% and from 14 to 64% for Caucasian women. The only instance, for which AA women had similar or lower 18-month % weight regain, was for those randomized to the self-directed maintenance arm of the TOURS study [15]. However, the sample size of women randomized to this treatment was relatively small, and results should be interpreted with caution. Cultural adaptations appeared to be an important component in multisite trials with a mixed race/ethnicity and gender sample as evidenced by less % weight regain for AA women in the TONE [44], DPP [11], and WLM [56] trials. Inclusion of a formal maintenance program was associated with lower % weight regain for AA and Caucasian women [3, 11, 13–15, 44, 50, 56, 60, 62, 65] compared to programs without a formal maintenance intervention [16, 61, 67, 70]. Lastly, weight maintenance for the WLM trial [56] was reported at 36-month follow-up only. Both AA and Caucasian women responded favorably to individualized sessions whereas AA women responded less favorably to the internet-based maintenance format. 

## 4. Discussion

This paper reports on a systematic review of the behavioral lifestyle intervention literature published between 1990 and 2011 that reported weight outcomes, included a maintenance phase of at least six months, and enrolled or specifically targeted AA women. Only 17 studies met the inclusion criteria, underscoring the limited research in this area. The studies reviewed differed in design, duration, and intensity of the maintenance interventions, sample size, and attrition rates, which led to the inevitable challenge of cross-study comparisons. 

Generally, AA women lost less weight during the intensive weight loss phase and maintained a lower % of their weight loss compared to Caucasian women in the behavioral lifestyle interventions reviewed [11, 13–15, 44, 65]. However, for studies reporting 18-month weight maintenance outcomes, in all but two [67, 70], AA women maintained some percentage of the weight loss achieved during the intensive weight loss phase. This is important given that small, sustained weight losses are associated with clinically meaningful health benefits [17, 18]. The TONE trial [44] was the only study in which AA women had similar weight loss and maintenance as Caucasian women. Importantly, the sample of AA women in the TONE trial was relatively small (*n* = 46), retention poorer than that for Caucasian women, and women were older, overweight/obese, and hypertensive. This may reflect a nonrepresentative and more motivated sample. 

The most remarkable finding was that the majority of studies failed to describe the specific strategies used in the delivery of the maintenance intervention, adherence to those strategies, and did not incorporate a maintenance phase process evaluation making it difficult to identify intervention characteristics associated with better weight control. Also, many of the studies did not report a distinction between what similar or different behaviors were performed during the active and maintenance phase of the intervention. This may be due to the fact that often, the active intervention phase does not lead to sufficient weight losses to warrant an active maintenance phase. Other than the WLM trial [56], a set amount of weight loss was not used as a criterion for participating in the maintenance phase of the other trials [3, 11, 13–16, 44, 50, 60–62, 65, 67, 69, 70]. Many individuals remain obese, even after one year of treatment, and continue to desire to lose [7]. Therefore, the maintenance phase, which is often arbitrarily set by the study investigators, may not truly reflect participants engaging in weight maintenance-type behaviors. Furthermore, behaviors associated with successful weight management such as monitoring of food intake [77], limited intake of fast food [76], and sugar sweetened beverages [58], limited TV viewing [78], regular self-weighing [79], eating breakfast [80], and meal planning [81] were not closely tracked or routinely reported or, when reported, distinctions were not made based on race/ethnicity and or sex [9]. In a recent article, by Barnes and Kimbro [82], limiting fat intake, consuming less fast food, and monthly weighing were associated with better long-term weight control in AAs who successfully reduced their weight by ≥10% and maintained the loss for at least one year. This further emphasizes that consistent documentation of these types of behaviors in the literature, and by race/ethnicity and sex when appropriate, can help to identify behaviors that lead to successful long-term weight control [83, 84].

Despite this significant caveat, we attempted to identify design components that influence the effectiveness of behavioral lifestyle interventions designed to promote weight maintenance specific to AA women. Findings suggest that inclusion of cultural adaptations may result in more favorable weight maintenance outcomes for AA women and is consistent with the existing literature [3, 45]. For example, in the multisite TONE [44], WLM [56], and DPP [11] trials, enrolling a mixed race/ethnicity and gender sample, inclusion of cultural adaptations resulted in superior weight outcomes compared to HPT [13], TOHP [13], and TOHP II [65] trials. However, it is hard to discern what specific cultural adaptations or combination of adaptations are most useful [71]. What researchers consider to be “salient” cultural adaptations is often derived from qualitative studies [85–87], based on community input [88], based on researcher perception of sociocultural perspectives of AAs, or, informal participant and community leader conversations [89]. For example, AA women have cited inclusion of spirituality as a culturally salient adaptation to promote weight control [90]. However, when tested empirically, in the trial by Djuric et al. [62], inclusion of spirituality counseling did not result in better weight outcomes. It may be that several rather than a single adaptation is necessary for a particular population or setting [91]. However, assessment and comparison of a package of cultural adaptations presents an empirical challenge [91, 92]. Nonetheless, a clearer definition of what constitutes a cultural adaptation and a better understanding of the mechanistic relationship between cultural adaptations and the weight control process are needed. 

Not surprising, inclusion of a formal maintenance program was largely associated with lower % weight regain for both AA and Caucasian women [3, 13–15, 44, 50, 56, 60, 62, 65] compared to studies lacking a formalized program [16, 61, 67, 70]. This finding is consistent with two other reviews investigating weight management in minority and nonminority populations [37, 93]. However, in two of these trials [3, 15], AA women randomized to the self-directed or no contact maintenance arm had lower % weight regain at 18-month follow-up [3, 15]. It is unclear why less contact resulted in better weight maintenance although the study authors speculated that the design, setting, or staffing [3] or a failure to culturally adapt the maintenance intervention [15] may have resulted in the outcomes observed. As for the more unique design features, AA and Caucasian women randomized to motivational interviewing had lower % weight regain compared to women allocated to the attention control counseling [14], whereas Internet delivery was less effective than personalized treatment, particularly for AA compared to Caucasian women, in the WLM trial [56]. A previous study found that randomization to internet maintenance resulted in greater weight regain as compared to in-person treatment [94]. Furthermore, at 12-month follow-up, 70% of internet participants reported that they would have preferred in-person contact [94], suggesting that *a priori* knowledge concerning an individual's acceptability of treatment delivery mode may increase an intervention's effectiveness.

African American and Caucasian women were more successful with weight maintenance when study participants were recruited for this purpose. It may be that AA women recruited for interventions where weight loss was secondary (e.g., walking intervention, sodium reduction) [67, 70] were less interested or motivated to lose weight. In a review of pretreatment predictors, self-motivation, general efficacy, and autonomy were all consistent pretreatment predictors of long-term weight success (1 year or more) [95]. Therefore, designing an intervention that places the priority on weight loss throughout the trial (i.e., from recruitment to implementation and maintenance phases) might improve weight outcomes. 

### 4.1. Limitations

Some limitations in our study deserve mention. We included RCTs, pilot RCTs, and nonrandomized controlled and single group design trials. The small sample sizes of the nonrandomized trials and higher attrition rates in several of the studies may have introduced selection bias [3, 11, 13, 14, 50, 61, 67, 69, 70]. Data obtained from study authors were for completers only which may have led to reporting bias [3, 16, 44, 50]. Similarly, many of the studies reported data from completers or persons with available follow-up data which could also lead to reporting bias. With a limited number of studies reporting racial/ethnic and sex differences, this paper did not fully capture differences in terms of the efficacy of behavioral lifestyle interventions on weight loss maintenance, among AA women [36]. The varying lengths of the maintenance periods may have also confounded the findings. Additional limitations include the exclusion of studies not published in English and of studies predating 1990. We also intentionally did not explore the differential effects of food provision, surgery, or pharmaceutical intervention's on weight loss maintenance in AA women. 

## 5. Conclusion and Future Directions

Overall, our synthesis of the literature shows that AA women struggle unduly with both weight loss and maintenance. All of the studies reviewed focused specifically on individual behavior change strategies. It may be that the inherent biology and social and environmental constraints of AA women, unfavorably impacts their adoption of these behaviors [45, 96]. In terms of biology, studies suggest that AA women have several metabolic and physiologic factors that may account for their difficulty with weight management. These factors include less energy expenditure when sleeping, exercising, and in the resting state [97, 98]; alterations in fat oxidation consistent with increased fat storage [99]; higher steady-state ghrelin levels which leads to increased hunger [100]; lower PYY production after meal which could lessen satiety [101]; and decreased energy cost of activity following diet-induced weight loss [98]. The biological aspects of weight regain are increasingly being studied and understood [102, 103]. However, future studies should examine these biological factors within the context of weight loss/maintenance trials and test for racial/ethnic differences.

In terms of AA women's socioenvironment, several factors may hinder their adoption of behaviors shown to positively impact weight control. These factors include socioeconomic status [104, 105], availability and access to high quality foods [106, 107], availability and access to PA resources [108, 109], heightened exposure to unhealthy foods [110], neighborhood safety [111], stress [111], discrimination [112], and dysfunctional social networks [113]. Behavioral economic research suggests that these intertwining biological and contextual factors place eating and PA behaviors beyond an individual's rational control [114]. Therefore, future research should evaluate how biologic and socioeconomic factors mediate diet and PA behavior change within a weight management trial. Additionally, researchers might attempt to understand these pathways prior to developing interventions and utilize findings to inform future intervention design. 

 The emergence of system-oriented and multilevel research will provide greater insight into the relational complexity of individual- and population-level factors affecting weight management [96]. Quantification of these factors' influence on weight control and identification of the optimum level for intervention within subgroups of the population pose a complex set of research questions for investigators [45]. Cross-disciplinary, translational research addressing the intersection between individual behaviors, biology, social, and environmental contextual factors will allow researchers to more effectively design and evaluate interventions that simultaneously address multiple mechanisms of weight management [96]. The ultimate goal of this research is to make the adoption of healthy eating and regular PA within everyday life the easier option [96]. Continued research that affords a more complete understanding of the complex connectedness of the behavioral, sociocultural, environmental, and biologic factors that lead to successful weight control in this population is warranted. 

## Figures and Tables

**Table 1:** Behavioral lifestyle interventions reporting weight maintenance outcomes for african american women (1990–2011) (*n* = 17).

Author and year of publication	Quality ranking score^†^	Study design, setting, and length of trial	Participant characteristics^††^	Maintenance phase characteristics	Frequency, delivery, and dose (time), of maintenance components	Mean baseline weight (kg) (±SD/SE)	Weight change following intensive intervention phase mean (±SD/SE) weight (kg)	Weight change (kg) from baseline during maintenance phase follow-up (SD/SE)	Adherence to maintenance sessions/components retention (%)
Kumanyika et al. (2002)^‡‡^ [44] Trials of Nonpharmacologic Intervention in the Elderly (TONE)	**11**	Design: RCT Setting: Academic Medical Centers Length of Trial: Up to 34 month	*n* = 585 (all overweight participants randomized to a weight loss treatment arm) *Weight loss/Sodium* AAW: 21 CW: 43 *Weight loss* AAW: 25 CW: 50 Mean Age (y): AAW: 65.5 (±4.8) CW: 65.8 (±4.5) Income: NDR Education: College graduate: AAW: 25.6% CW: 41.8% Health status: Hypertensive BMI > 27.8 kg/m^2^	Formal Theoretical Framework: SCT Cultural Adaptations: Yes, attention to cultural diversity, staff training, and printed materials Duration of maintenance phase: 7–28 months (varied by participant) Criteria for entry into WL Maintenance Phase: None Components Targeted at WL Maintenance: Didactic nutrition PA (self) Behavioral Modification Strategies (i) Self-monitoring of food intake, food-related behaviors, and PA (ii) Goal-setting Problem solving Relapse prevention [55]	Frequency, Delivery, and Dose: *Months 7-*8 Biweekly group or individual session Dose: 60 min (4 sessions total) *Months 9+* Monthly group or individual sessions Dose: 60 min (up to 17 sessions) Monthly phone or mail-based contact (up to 17 contacts) [55]	*Weight loss/Sodium* AAW: 84.0 (±6.9) kg CW: 82.7 (±9.7) kg *Weight loss* AAW: 82.9 (±9.3) kg CW: 82.3 (±9.0) kg	*Weight loss/Sodium* AAW: −3.9 (±3.6) kg CW: −3.9 (±3.9) kg *Weight loss* AAW: −3.3 (±2.8) kg CW: −5.8 (±3.5) kg	*12 month Fu* Weight loss/Sodium AAW: −4.0 kg CW: −3.7 kg Weight loss AAW: −3.9 kg CW: −5.9 *18 month Fu* *Weight loss/Sodium* AAW: −3.6 kg CW: −3.3 kg *Weight loss* AAW: −3.8 kg CW: −5.0 kg *Proxy for end of trial (24 month Fu) Weight loss/Sodium* AAW: −2.6 kg CW: −2.7 kg *Weight loss* AAW: −3.5 kg CW: −4.6 kg	Adherence to maintenance sessions/components: NDR Retention: *Weight loss/Sodium* AAW: 62% CW: 88% *Weight loss* AAW: 48% CW: 66%

Kumanyika et al. (2005)^‡^ [3] Healthy Eating and Lifestyle Program (HELP)	**11**	Design: RCT Setting: Academic Medical Center Length of Trial: 15 or 21 months (cohort dependent)	AAW: *n* = 116 (*n* = 87 AAW Phase 2 completers) *Group HELP maintenance* AAW: *n* = 24 Mean Age (y): 47.4 (±11.1) Education: >HS = 80% *Self-HELPmaintenance* AAW: *n* = 24 Mean Age (y): 46.2 (±8.6) Education: >HS = 61% *Clinic only* AAW: *n* = 28 Mean Age (y): 46.1 (±10.1) Education: >HS = 67% Income: NDR Health status: BMI 30–50 kg/m^2^ Medically cleared	Formal Theoretical Framework: SCT Cultural Adaptations: Yes, study logo; adapted materials specific to AA; inclusion of AA interventionist Duration of maintenance phase: 18 months (cohorts 1 and 2) 12 months (cohorts 3 and 4) Criteria for entry into WL Maintenance Phase: Attended postphase 1 data collection Components Targeted at WL Maintenance: Didactic Nutrition PA (self and occasional S weekend group walks) Behavioral Modification Strategies (i) Self-monitoring of food intake and PA (ii) Goal-setting (iii) Problem solving (iv) Relapse prevention	Frequency, Delivery, and Dose: *Group HELP maintenance* *Months 7*–*9* biweekly group class Dose 60 min (6 total classes) * Months 10+* once monthly group class Dose: 60 min (8 classes total for cohorts 1 and 2; 3 classes total for cohorts 3 and 4) Group walking held occasionally Individualized nutrition, PA or behavioral consultations upon request 3 clinic visits (cohorts 1 and 2) 2 clinic visits (cohorts 3 and 4) *Self *-*HELP* *Maintenance* Self-help kit (local restaurant and fitness guide, diaries, pedometer) 1 in-person group meeting Teams formed to promote peer support Once monthly call Group walking held occasionally 3 clinic visits (cohorts 1 and 2) 2 clinic visits (cohorts 3 and 4) *Clinic only* 3 clinic visits (cohorts 1 and 2) 2 clinic visits (cohorts 3 and 4)	100.8 (±15.9) kg	*Group HELP *maintenance −1.6 kg (±3.3) kg *Self-HELP maintenance* −2.0 (±4.1) kg *Clinic only* −1.6 (±3.7) kg	*12 month Fu* NDR * End of Trial (12 or 18 month Fu depending on cohort)* *Group HELP maintenance* −0.8 (±4.4) kg * Self-HELP maintenance* −1.3 (±5.5) kg * Clinic only* −1.4 (±5.7) kg	Adherence to maintenance sessions/components: * Group HELP* Mean attendance 40% at biweekly classes Mean attendance 31% at monthly classes *Self-HELP* 35–55% of participants were successfully reached for the monthly phone-based contact Retention: 66% (all treatments)

Svetkey et al. (2008)^‡‡^ [56] Weight Loss Maintenance Trial (WLM) Post-intervention weight change crudely calculated from data presented in the manuscript	**11**	Design: RCT Setting: Academic Medical Centers Length of Trial: 36 months	*n* = 1032 (Randomized to phase II) *Self-directed maintenance* AAW: 90 CW: 131 *Technology maintenance* AAW: 90 CW: 130 *Personal contact maintenance* AAW: 87 CW: 126 Mean Age (y): AAW: 53 (±9.0) CW: 57 (±9.0) [8] Income: AAW: 42% ≥ $60,000/y CW: 71% ≥ $60,000/y [8] Education: College degree or higher: AAW: 56% CW: 72% [8] Health status: BMI 25–45 kg/m^2^ Hypertensive and/or dyslipidemic	Formal Theoretical Framework: SCT Cultural Adaptations: Yes, Minority Implementation committee, AA cultural-training for all interventionists, cultural sensitivity training, development of specific strategies for enhancing intervention effectiveness for AA [57] Duration of maintenance phase: 30 months Criteria for entry into WL Maintenance Phase: ≥4 kg loss during IWL phase Components Targeted at WL Maintenance: Continue calorie control/DASH diet pattern Didactic Nutrition PA (self, goal: 225 min/wk) Behavioral Modification Strategies (i) Self-monitoring, food intake, weight, and PA (ii) Problem-solving (iii) Goal-setting Relapse prevention MI	Frequency Delivery, and Dose: *Self-directed maintenance* One individual in-person session and printed materials *Technology maintenance* Unlimited access to an interactive web-site Dose: encouraged to log on at least once per week *Personal contact maintenance* Once monthly individual in-person or phone-based sessions Dose: 5–15 minutes and every 4th month 45–60 minutes (30 sessions total)	AAW: 94.8 (±15.2) kg CW: 89.5 (±15.2) kg	AAW: −7.1 kg CW: −8.0 kg	*12 month Fu* NDR *18 month Fu* NDR End of Trial (36 month Fu) Self-directed maintenance* (based on adjusted values) AAW: −1.8 (se = 0.6) kg CW: −2.2 (se = 0.6) kg Technology maintenance* (based on adjusted values) AAW: −1.3 (se = 0.6) kg CW: −3.0 (se = 0.6) kg Personal contact maintenance* (based on adjusted values) AAW: −2.2 (se = 0.6) kg CW: −3.9 (se = 0.6) kg	Adherence to maintenance sessions/components: *Self-directed maintenance* NA *Technology maintenance* 77% logged on at least 1/week *Personal contact maintenance* 91% of attendance at offered sessions Self-report frequency weighing/week AAW: 3.0 (0.1) CW: 2.6 (0.1) Retention: *Self-directed maintenance* 94% (all participants) *Technology maintenance* 93% (all participants) *Personal contact maintenance* 94% (all participants)

West et al. (2008) [11] Diabetes Prevention Program (DPP) Data reported for the IL arm only	**11**	Design: RCT Setting: Academic Medical Centers Length of Trial: 36 months	*n* = 2921 *IL intervention arm* AAW: 120 CW: 381 Age (y): AAW: 77.4% > 40 y CW: 75.6% > 40 y Income: NDR Education: NDR Health status: Impaired glucose tolerance	Formal Theoretical Framework: SCT Cultural Adaptations: Yes, case managers of same ethnic group, print materials tailored for ethnic group, literacy adaptations Duration of Maintenance phase: 24 months Criteria for entry into WL Maintenance Phase: None Components Targeted at WL Maintenance: Continued adherence to Fat and Calorie Control Didactic Nutrition PA (S and self: goal, 150 min/wk) Behavioral Modification Strategies (i) Self-monitoring food intake and PA (ii) Goal-setting Individual “Tool-box”	Frequency, Delivery, and Dose: At least one bimonthly individual, in-person session Dose: 15–45 minutes Contacted at least once by phone in between sessions (However, coaches could meet with individuals as often as needed) Group-based courses (3/year) Maintenance campaigns to promote adherence (3-4/year) [59]	AAW: 82.0 (±14.8) kg CW: 95.1 (±21.2) kg	AAW: −4.7 (±5.1) kg CW: −7.5 (±5.6) kg	*12 month Fu* AAW: −4.4 ± 6.0 kg CW: −7.8 ± 7.4 kg *18 month Fu* AAW: −3.9 ± 6.1 kg CW: −6.6 ± 8.2 kg *End of maintenance phase (36 month Fu)* AAW: −2.1 ± 6.3 kg CW: −4.2 ± 7.5	Adherence to maintenance sessions/components: Mean 50.3 (±21.8) sessions (*all IL participants*) [58] Retention: AAW: 64% CW: 69%

Kumanyika et al. (2009)^‡^ [50] Supporting Healthy Activity and Eating Right Everyday study (SHARE)	**11**	Design: RCT Setting: Academic Medical Center Length of Trial**:** 24 Months	*Family High Support* AAW: *n* = 62 Mean Age (y): 47.3 (±7.3) Income: NDR Education: >HS = 86% *Family Low Support* AAW: *n* = 57 Mean Age (y): 50.2 (±8.2) Income: NDR Education: >HS = 77% *Individual High Support* AAW: *n* = 29 Mean Age (y): 48.2 (±7.7) Income: NDR Education: >HS = 83% *Individual Low Support* AAW: *n* = 29 Mean Age (y): 46.8 (±6.6) Income: NDR Education: >HS = 71% Health status: Healthy or medically cleared	Formal Theoretical Framework: NDR Cultural Adaptations: Yes, AA program counselors, culturally-based content, community-based field trips Duration of maintenance phase: 18 months Criteria for entry into WL Maintenance Phase: None Components Targeted at WL Maintenance: Calorie control Didactic Nutrition PA (S and self, goal 180 min/wk) Behavioral Modification Strategies (i) Self-monitoring PA (ii) Problem solving	Frequency, Delivery, and Dose: *Months 7*–*1*2 Biweekly groups sessions Dose: 90 minutes 2-3 in-person individual sessions Dose: 45–60 minutes (14-15 session total) *Months 13*–*24* Once monthly group sessions Dose: 90 minutes Three in-person individual sessions Dose: 45–60 (15 sessions total) Quarterly newsletter (6 newsletters)	*Family High Support* 103.1 (±11.3) kg *Family Low Support* 106.5 (±16.3) kg *Individual High Support* 102.9 (±21.2) kg *Individual Low Support* 97.3 (±16.1)	*Family High Support* −5.1 (±4.4) kg *Family Low Support* −5.0 (±4.8) kg *Individual High Support* −3.8 (±5.4) kg *Individual Low Support* −3.4 (±4.1)	*12 month Fu* *Family High Support* −5.9 (±5.2) kg *Family Low Support* −6.4 (±6.5) kg *Individual High Support* −4.4 (±5.6) kg *Individual Low Support* −2.1 (±3.7) kg *18 month Fu* *Family High Support* −4.8 (±6.7) kg Family Low Support −5.1 (±6.3) kg *Individual High Support* −3.6 (±7.0) kg *Individual Low Support* −3.0 (±3.6) kg *End of Trial (24 month Fu)* *Family High Support* −3.0 (±6.1) kg *Family Low Support* −3.1 (±6.6) kg *Individual High Support* −1.1 (±7.23) kg *Individual Low Support* −3.2 (±6.4) kg	Adherence to maintenance sessions/components: *Months 7–12* *Group* *Sessions* Median 0–4 sessions attended across treatments *Individual sessions* Median 0-1 session across treatments *Months 13–24* *Group sessions* Median 0 sessions attended across treatments *Individual sessions* Median 0 sessions attended across treatments Retention: *Family High Support* 66% *Family Low Support* 68% *Individual High Support* 69% *Individual Low Support* 55%

Fitzgibbon et al. (2010) [60]	**11**	Design: RCT Setting: University Length of Trial: 18 months	*n* = 213 I: 107 AAW C: 106 AAW Mean Age (y): I: 46.4 (±8.4) C: 45.5 (±8.4) Median Income: $42,500/y Education (y): I: 14.6 (±2.0) C: 15.1 (±1.9) Health status: BMI 30–50 kg/m^2^ Healthy or medically cleared (Disease prevalence: NDR)	Formal Theoretical Framework: SCT Cultural Adaptations: Yes, attention to food and activity cultural preferences, AA peer mentors, religion and spirituality intertwined into messaging. Duration of maintenance phase: 12 months Criteria for entry into WL Maintenance Phase: None Components Targeted at WL Maintenance: General calorie fat control, increased fiber, FV	Frequency, Delivery, and Dose: *Months 7–12* Twice weekly group S PA Dose: 60 minutes Once weekly didactic session (took place prior to one of the S PA sessions) Dose: 30 minutes (48 sessions total) Once monthly MI session Dose: 20–30 minutes (6 sessions total)	I: 104.3 (±15.6) kg C: 105.8 (±17.8) kg	I: −3.0 (±4.9) kg C: +0.2 (±3.7) kg	*12 month Fu* NDR *End of maintenance* *(18 month Fu)* I: −2.3 (±7.4) kg C: +0.5 (±4.7) kg	Adherence to maintenance sessions/components: Percentage of maintenance classes attended = 27% 30% of participants attended at least half of the offered maintenance classes Retention: I: 87% C: 92%

Martin et al. (2008)^‡‡^ [61]	**10**	Design: RCT Setting: Community Clinic Length of Trial: 18 months	I: 68 AAW C: 69 AAW Mean Age (y): I: 40.8 (±12.7) C: 42.6 (±11.4) Income: <$16,000/y Education: Graduated HS I: 83% C: 74% Health status: Healthy and medically cleared (Disease prevalence: NDR)	Formal Theoretical Framework: NDR Cultural Adaptations: Yes, menus and recipes books Duration of maintenance phase: 12 months Criteria for entry into WL Maintenance Phase: None Components Targeted at WL Maintenance: Self-directed	Frequency, Delivery, and Dose: Three clinic visits for follow-up assessments by research staff (I and C) Dose: NDR	I: 101.2 (±20.6) kg C: 103.4 (±18.0) kg	I: −1.4 kg C: +0.3 kg	*12 month Fu* I: −1.4 (±3.7) kg C: −0.3 (±3.6) kg *End of Trial (18 month Fu)* I: −0.5 (±3.3) kg C: +0.1 (±3.8) kg	Adherence to maintenance sessions/components: NDR Retention: 63% (I and C)

Djuric et al. (2009) [62] Weight change at end of trial crudely calculated from data presented in the manuscript	**10**	Design: RCT, pilot Setting: University Length of Trial: 18 months	I (*spirituality and dietary counseling maintenance*): 12 AAW C (dietary counseling only maintenance): 12 AAW Mean Age (y): I: 55.0 C: 56.0 Income: I: <$30,000/year = 25% C: <$30,000/year = 25% Education: I: College graduate: 67% C: College graduate: 50% Health status: Breast cancer survivors BMI 30–45 kg/m^2^	Formal Theoretical Framework: SCT Cultural Adaptations: Yes, spirituality Duration of maintenance phase: 12 months Criteria for entry into WL Maintenance Phase: None Components Targeted at WL Maintenance: Calorie/Portion and Fat Control Didactic Nutrition PA (self, goal 150 min/wk) Behavioral Modification Strategies (i) Self-monitoring of food intake and activity Spirituality counseling (I only)	Frequency, Delivery, and Dose: *I (spirituality and dietary counseling maintenance*) *Months 7–18* Dietary counseling, 1 individual in-person session at month 12, otherwise once monthly phone based sessions Dose: NDR (12 sessions total) *Months 7–9* Spirituality counseling Once weekly individual phone-based sessions (up to 12 sessions) *ߙ* *Months 10–12* biweekly individual phone-based sessions (up to 6 sessions) *Months 13–18* Once monthly individual phone-based sessions (up to 6 sessions) Dose: 17–45 minutes/call C (dietary counseling only maintenance) *Months 7–18* Dietary Counseling, one individual in-person session at month 12, otherwise once monthly phone based sessions Dose: NDR (up to 12 sessions) I and C: *Months 7*–*18 *once monthly mailed newsletter (12 newsletters)	I: 93.8 (±11.3) kg C: 94.9 (±14.8) kg	I: −1.0 (±6.5) kg C: −2.6 (±5.1) kg	*12 month Fu* NDR *End of Trial (18 month Fu)* I: −0.7 kg C: −2.2 kg	Adherence to maintenance sessions/components: *I (spirituality and dietary counseling maintenance*) Spirituality counseling calls ranged from 2–26 completed per participant Retention: 92% (all participants)

Kumanyika et al. (1991) [13] Hypertension Prevention Trial (HPT)	**9**	Design: RCT Setting: Academic Medical Centers Length of Trial: 36 months	*n* = 236 (weight loss tx arms only) AAW: 28 CW:43 Age (y): 25–49 (all participants) Income: NDR Education: College graduate: 48% (all participants) Health status: Healthy, normotensive	Formal Theoretical Framework: NDR Cultural Adaptations: NDR Duration of maintenance phase: 30 months Criteria for entry into WL Maintenance Phase: None Components Targeted at WL Maintenance: Didactic Nutrition Behavioral Modification Strategies	Frequency Delivery, and Dose: *Months 7*–*36 *Bimonthly individual/group sessions Dose: NDR (15 sessions offered) Bimonthly mailed newsletter (15 newsletters sent) [63]	AAW: 77.2 (±9.9) kg CW: 78.0 (±10.9) kg	AAW: −2.6 (±3.9) kg CW: −4.7 (±4.3) kg	*12 month Fu* AAW: −1.4 ± 2.9 kg CW: −3.3 ± 5.7 kg *18 month Fu* AAW: −0.03 ± 4.7 kg CW: −1.7 ± 5.8 kg *End of Trial (36 month Fu)* AAW: +2.6 ± 4.7 kg CW: −1.2 ± 7.2 kg	Adherence to maintenance sessions/components: NDR Retention: AAW: 93% CW: 93%

Kumanyika et al. (1991) [13] Trials of Hypertension (TOHP)	**9**	Design: RCT Setting: Academic Medical Centers Length of Trial: 18 months	*n* = 303 (weight loss arms only) AAW: 33 CW: 48 Age (y): 30–54 (all participants) Income: NDR Education: College graduate: 50% (all participants)	Formal Theoretical Framework: NDR Cultural Adaptations: NDR Duration of maintenance phase: 12 months Criteria for entry into WL Maintenance Phase: None Components Targeted at WL	Frequency, Delivery, and Dose: Varied by participant but could include one or a combination of: (a) monthly informal group sessions (b) group weigh-in (c) individual weigh-in (d) individual counseling Dose: NDR [64]	AAW: 79.9 (±10.0) kg CW: 79.7 (±10.8) kg	AAW: −1.9 (±3.5) kg CW: −4.9 (±4.8) kg	*12 month Fu* AAW: −1.1 ± 4.1 kg CW: −3.6 ± 5.2 kg *End of Trial (18 month Fu)* AAW: −0.02 ± 4.1 kg CW: −2.5 ± 6.3 kg	Adherence to maintenance sessions/components: 90% participation (including make-up, all participants) Retention: AAW: 97% CW: 100%

Stevens et al. (2001) [65] Trial of Hypertension Prevention II (TOHPII)	**9**	Design: RCT Setting: Academic Medical Centers Length of Trial: 36 months	I: 64 AAW C: 49 AAW Mean Age (y): I: 43.4 (±6.1) (all participants) C: 43.3 (±6.1) (all participants) Income: NDR and Education: NDR Health Status: Systolic BP < 140 mmHg Diastolic BP 83–89 BMI: 24.4 to 37.4 kg/m^2^ (all women)	Formal Theoretical Framework: NDR Cultural Adaptations: NDR Duration of maintenance phase: 32 months Criteria for entry into WL Maintenance Phase: None Components Targeted at WL Maintenance: Didactic Nutrition PA (SD) (30–45, four to five days per week) Behavioral Modification Strategies (i) Goal-setting (ii) Problem-solving (iii) Self-monitoring of food intake and PA	Frequency, Delivery, and Dose: [66] Months 5–7: biweekly group sessions 6 biweekly group sessions Months 8–1. 7: once monthly group sessions Months 18+: biweekly individual contact (phone, face to face, and mail) Attendance at 3 of 6 minimodules yearly (each module was 3–6 group session)	I: 84.1 (±11.9) kg (all women) C: 82.9 (±10.9) kg (all women)	I: AAW: −2.1 (CI: −3.0 to −1.3) kg CW: −3.6 (CI: −4.4 to −2.8) kg C: AAW: +0.3 (CI: −0.6 to +1.2) kg CW: +0.2 (CI: −0.4 to +0.7) kg	*12 monthFu* NDR *18 month Fu* I: AAW: −0.4 (CI: −1.6 to 0.9) kg CW: −1.7 (CI: −2.6 to −0.7) kg C: AAW: +0.4 (CI: −0.8 to 1.6) kg CW: 0.4 (CI: −0.3 to 1.2) kg *36 month Fu* I: AAW: +0.5 (CI: −1.1 to 2.0) kg CW: 0.8 (CI: 0.3 to 1.9) kg C: AAW: +1.7 (CI: 0.2 to 3.1) kg CW: 1.4 (CI: 0.3 to 2.5) kg	Adherence to maintenance sessions/components: Months 6–18: median sessions attended, 11 Months 19–36**: **median sessions attended, 7.5 Retention: I: AAW: 97% CW: 98% C: AAW: 100% CW: 97%

Yancey et al. (2006) [67]	**9**	Design: RCT Setting: Community, Urban Length of Trial: 12 months	*n* = 366 AAW I: 188 C: 178 Mean Age (y): I: 58.0 (±0.9) C: 60.1 (±0.5) Income: I: $40,000–59,000 C: $40,000–59,000 Education (y): I: 15.06 (±2.16) C: 14.98 (±2.24) Health status: NDR	Formal Theoretical Framework: Social Ecological Model Cultural Adaptations: Yes, trial specific to black women, chosen study site, AA instructors Duration of maintenance period: 10 months Criteria for entry into WL Maintenance Phase: None Components Targeted at WL Maintenance: Self-directed Free fitness club membership (I and C)	Frequency, Delivery, and Dose: No contact	I: 81.5 kg (*n* = 92) C: 82.7 kg (*n* = 79)	I: +0.05 kg C: +0.3 kg	*End of maintenance* *(12 month Fu)* I: +1.4 kg C: +1.02 kg	Adherence to maintenance sessions/components: NDR Retention: I: 72% C: 72%

West et al. (2007) [14] Weight change crudely extrapolated from Figure 1 in the manuscript	**9**	Design: RCT Setting: University Length of Trial: 18 months	*MI group* AAW: 43 CW: 66 *Attention control group* AAW: 41 CW: 67 Mean Age (y): 53 ± 10 (all participants) Education: College education or higher: 35% (all participants) Income: NDR Health Status: Type 2 Diabetes (no insulin use) BMI 27–50 kg/m^2^	Formal Theoretical Framework: NDR Cultural Adaptations: NDR Duration of maintenance phase: 12 months Criteria for entry into WL Maintenance Phase: None Components Targeted at WL Maintenance: Calorie and fat control Didactic Nutrition PA (self, goal 150 min/wk) Behavioral Modification Strategies (i) Goal-setting (ii) Problem-solving (iii) Self-monitoring of food intake and PA (iv) Stimulus control (v) Relapse prevention MI or Attention Control sessions	Frequency, Delivery, and Dose: *Months 7–12 *Biweekly group sessions Dose: NDR (12 sessions total) *Months 7*–*12* Two individual MI or Attention Control sessions Dose: 45 minutes per session (5 sessions total) *Months 13*–*18* Once monthly group session Dose: NDR (6 sessions total)	*MI group* 97 (±17) kg (all participants) *Attention control group* 97 (±15) kg (all participants)	*MI group* AAW: −3.4 kg CW: −5.3 kg *Attention control group* AAW: −2.9 kg CW: −3.4 kg	*12 month Fu* *MI group* AAW: −2.9 kg CW: −5.9 kg *Attention control group* AAW: −1.8 kg CW: −3.3 kg *End of maintenance phase (18 month Fu)* *MI group* AAW: −1.9 kg CW: −4.4 kg *Attention control group* AAW: −1.0 kg CW: −2.0 kg	Adherence to maintenance sessions/components: *Group sessions* *7*–*12 months* 57% attendance *13*–*18 months* 48% attendance *Food diaries submitted* *7–12 months* 7 ± 9 diaries *13*–*18 months* 5 ± 9 diaries Retention: 93% (all participants)

Rickel et al. (2011)^‡‡^ [15] Treatment of Obesity in Underserved Rural Settings (TOURS) Weight change crudely calculated from data presented in the manuscript	**9**	Design: RCT Setting: Community, rural Length of Trial: 18 months	*n* = 234 AAW: 43 CW: 181 Mean Age (y): AAW: 58.0 (±0.9) CW: 60.1 (±0.5) Income: AAW: <$50,000/y: 70% CW: <$50,000/y: 66% Education: High school degree or less AAW: 28% WW: 39% Health status: BMI > 30.0 kg/m^2^	Formal Theoretical Framework: NDR Cultural Adaptations: Southern-focused Duration of maintenance phase: 12 months Criteria for entry into WL Maintenance Phase: None Components Targeted at WL Maintenance: Calorie control Didactic Nutrition PA (self, goal 30 min/day walking) Behavioral Modification Strategies (i) Problem solving (ii) Self-monitoring [68]	Frequency, Delivery, and Dose: All treatment groups received handouts describing how to use problem solving to deal with obstacles related to WL maintenance *Extended care maintenance* *Phone-based counseling* Biweekly individual phone-based counseling sessions Dose: 15–20 minutes (26 sessions) OR *Face to Face counseling* Biweekly in-person group session Dose: 60 minutes (26 sessions)	AAW: 99.9 (±2.6) kg CW: 95.8 (±1.1)	AAW: −6.8 (±0.80) kg CW: −10.7 (±0.38) kg	*12 month Fu* NDR *End of Trial (18 month Fu)* *Extended care maintenance* AAW: −4.9 kg CW: −9.2 kg *Control * AAW: −5.5 kg CW: −6.5 kg	Adherence to maintenance sessions/components: *Record keeping (hours)* *Phone-based* 16.0 (±18.1) hours *Face to face* 15.7 (±18.9) hours *Control* 10.4 (±15.7) hours *Counseling time* *Phone-based* 10.2 (±12.4) hours *Face to face* 21.3 (±16.0) hours *Control* NA Retention: 96% (all participants)

McNabb et al. (1993) [69]	**7**	Design: NRCT, pilot Setting: Community Clinic, Urban Length of Trial: 12 months	*n* = 23 I: 13 AAW C: 10 AAW Mean Age (y): I: 57 C: 62 Income: NDR Education: I: Completed HS: 89% C: Completed HS: 85% Health status: Type 2 Diabetes 120% IBW	Formal Theoretical Framework: NDR Cultural Adaptations: Yes, trial specific to AA women Duration of maintenance period: 7.5 months Components Targeted at WL Maintenance: Self-directed Criteria for entry into WL Maintenance Phase: None	Frequency, Delivery, and Dose: No contact	I: 93.5 (±17.8) kg C: NDR	I: −4.1 kg C: NDR	*End of trial (12 month Fu)* I: −4.4 kg C: +1.4 kg	Adherence to maintenance sessions/components: NDR Retention: I: 77% C: 100%

Tsai et al. (2010)^‡^ [16]	**7**	Design: RCT, pilot Setting: University Clinic, Urban Length of Trial: 12 months	*n* = 50 (*n* = 44 women) I: AAW: 18 C: AAW: 19 Mean Age (y): AAW: 48.3 (±12.8) Income: NDR	Formal Theoretical Framework: NDR Cultural Adaptations: NDR Duration of maintenance phase: 6 months	Frequency, Delivery, and Dose: 2 in-person PCP visits Dose: 2-3 minutes devoted to discussing weight control (I and C)	I: AAW: 98.7 ± 16.4 kg CW: 76.2 ± 10.3 kg C: AAW: 99.6 ± 14.3 kg CW: 100.9 ± 20.0 kg	I: AAW: −4.5 kg CW: −6.6 kg C: AAW: +0.5 kg CW: +2.1 kg	*End of trial (12 month Fu):* I: AAW: −1.6 kg CW: −3.2 kg C: AAW: −0.2 kg CW: −1.1 kg	Adherence to maintenance sessions/components: NDR Retention: 94% (all participants)

Banks-Wallace (2007) [70] Weight change crudely calculated from Table 1 in the manuscript	**5**	Design UCT, pilot Setting NDR Duration of Trial: 18 months	*n* = 21 AAW Mean Age (y): 50.3 Income: 62% < $24,000 Education: Completed HS: 100% Health status: Hypertensive	Formal Theoretical Framework: NDR Cultural Adaptations: Yes, trial specific to AA women Duration of Maintenance Phase: 6 months Criteria for entry into WL Maintenance Phase: None Components Targeted at WL Maintenance: Self-directed	Frequency, Delivery, and Dose: No contact	93.7 (±13.1) kg	−8.5 kg	*End of trial (18 month Fu)* +11.7 kg	Adherence to maintenance sessions/components: NDR Retention: 71%

^†^Total quality ranking score = [(Design: RCT = 4; pilot RCT = 3; nonrandomized controlled trial = 2; single group design = 1) + (Primary intervention focus on weight control: 1 = No; 2 = Yes) + (Inclusion of a formal maintenance program: 1 = No; 2 = Yes) + (Cultural Adaptations: 1 = no adaptations; 2 = limited to recruitment of AA participants; 3 = studies reporting adapting intervention-related content and other adaptations such as cultural sensitivity staff training)].

^††^Data reported for AAW or CW only unless indicated otherwise.

^‡^Weight change by sex/ethnicity obtained from main study author for AAW completers only.

^‡‡^Intention to treat or multiple imputations analysis.

AAW: african american women; BMI: body mass index; BP: blood pressure; C: control; CI: confidence interval; CW: caucasian women; DASH: dietary approaches to stop hypertension; FU: follow-up; HS: high school; I: intervention; IBW: ideal body weight; IL: intensive lifestyle; MI: motivational interviewing; NA: not applicable; NDR: no data reported; NRCT: non-randomized controlled trial; PA: physical activity; PCP: primary care physician; S: supervised; SD: standard deviation; SCT: social cognitive theory; SE: standard error; UCT: uncontrolled trial; WL: weight loss; Y: years.

**Table 2:** Quality Rankings, Total Quality Score, Maintenance Phase Characteristics, and % Weight Loss Regained at Follow-up for African American Woman Enrolled in US Behavioral Lifestyle Interventions, 1990–2011 (*n* = 17)*.

Author		Year	Maintenance group	Study design	Primary focus on weight control	Formal maintenance program	Cultural adaptations	Total quality score	Maintenance format	Frequency of maintenance sessions	% Weight regain 12 M^†^	% Weight regain 18 M^†^	% Weight regain > 18 M^†, ††^
Kumanyika et al. [44]		2002	Weight Loss/Sodium Reduction	4	2	2	3	11	Group and individual sessions	Biweekly then monthly	AAW: 0% CW: 6%	AAW: 7% CW: 17%	AAW: 33% CW: 30%
Kumanyika et al. [44]		2002	Weight loss	4	2	2	3	11	Group and individual sessions	Biweekly then monthly	AAW: 0% CW: 0%	AAW: 0% CW:13%	AAW: 0% CW: 21%
Kumanyika et al. [3]		2005	Group HELP	4	2	2	3	11	Group	Biweekly then monthly		49%	
Kumanyika et al. [3]		2005	Self HELP	4	2	2	3	11	Self-directed, 1 group session, some staff phone support	Infrequent		35%	
Kumanyika et al. [3]		2005	Clinic only	4	2	2	3	11	2-3 clinic visits only	Semi-annually		12%	
Svetkey et al. [56]		2008	Personal Contact	4	2	2	3	11	Individual session	Monthly			AAW: 66% CW: 49%
Svetkey et al. [56]		2008	Internet	4	2	2	3	11	Web-based	Weekly login			AAW: 80% CW: 64%
Svetkey et al. [56]		2008	Self	4	2	2	3	11	No contact	NA			AAW: 77% CW: 71%
West et al. [11]		2008	IL	4	2	2	3	11	Individual	At least monthly	AAW: 6% CW: 0%	AAW: 17% CW: 12%	AAW: 55% CW: 44%
Kumanyika et al. [50]		2009	Family High Support	4	2	2	3	11	Group and individual sessions	Biweekly then monthly	0%	6%	41%
Kumanyika et al. [50]		2009	Family Low Support	4	2	2	3	11	Group and individual sessions	Biweekly then monthly	0%	0%	40%
Kumanyika et al. [50]		2009	Individual High Support	4	2	2	3	11	Group and individual sessions	Biweekly then monthly	0%	5%	71%
Kumanyika et al. [50]		2009	Individual Low Support	4	2	2	3	11	Group and individual sessions	Biweekly then monthly	35%	12%	6%
Fitzgibbon et al. [60]		2010	Intervention	4	2	2	3	11	Group and individual session	Twice weekly, weekly, then monthly		33%	
Martin et al. [61]		2008	Intervention	4	2	1	3	10	No contact	NA	0%	64%	
Djuric et al. [62]		2009	Diet and Spirituality	3	2	2	3	10	Individual sessions	Weekly then biweekly		30%	
Djuric et al. [62]		2009	Diet only	3	2	2	3	10	Individual sessions	Monthly		15%	
Kumanyika et al. [13]		1991	Weight loss treatment arms	4	2	2	1	9	Group and individual sessions	Bimonthly	AAW: 46% CW: 30%	AAW: 88% CW: 64%	AAW: 215% CW: 74%
Kumanyika et al. [13]		1991	Weight loss treatments arms	4	2	2	1	9	Group and individual sessions	Monthly	AAW: 42% CW: 27%	AAW: 89% CW: 49%	
Stevens et al. [65]		2001	Intervention	4	2	2	1	9	Group and individual session/mail and phone-based contact	Biweekly then monthly		AAW: 81% CW: 53%	AAW: 123% CW: 122%
Yancey et al. [67]^‡^		2006	Intervention	4	1	1	3	9	No contact, free gym membership	NA		NA	
West et al. [14]		2007	MI	4	2	2	1	9	Group and individual sessions	Biweekly then monthly	AAW: 15% CW: 0%	AAW: 44% CW: 17%	
West et al. [14]		2007	Attention control	4	2	2	1	9	Group and individual sessions	Biweekly then monthly	AAW: 34% CW: 4%	AAW: 66% CW: 38%	
Rickel et al. [15]		2011	Extended Care	4	2	2	1	9	Individual sessions	Biweekly		AAW: 28% CW: 14%	
Rickel et al. [15]		2011	Self	4	2	2	1	9	Newsletter only	Biweekly		AAW: 19% CW: 39%	
McNabb et al. [69]		1993	Intervention	2	2	1	2	7	No contact	NA	0%		
Tsai et al. [16]		2010	Intervention	3	2	1	1	7	Two visits with PCP	Quarterly	AAW: 64% CW: 52%		
Banks-Wallace [70]		2007	Intervention	1	1	1	2	5	No contact	NA		138%	

*Total quality ranking score criteria: Study Design: RCT = 4; pilot RCT = 3; nonrandomized controlled trial = 2; single group design = 1; Primary intervention focus on weight control: 1 = No; 2 = Yes; Inclusion of a formal maintenance program: 1 = No; 2 = Yes; Cultural Adaptations: 1 = no adaptations; 2 = limited to recruitment of AA participants; 3 = studies reporting adapting intervention-related content and other adaptations such as cultural sensitivity staff training.

^†^% Weight change at follow-up time-points crudely calculated from data provided in the manuscripts.

^††^Svetkey et al., 2008 [56], % weight regain reported for 36-month follow-up; Kumanyika et al., 1991 [13], % weight regain reported for 36-month follow-up; Kumanyika et al., 2002 [44], % weight regain reported for 24-month follow-up; Kumanyika et al., 2009 [50], % weight regain reported for 24-month follow-up; Stevens et al., 2001 [65], % weight regain reported for 36-month follow-up.

^‡^No weight loss achieved during intensive intervention phase, therefore, no weight regain to report.

AAW: African American Women; CW: Caucasian Women; IL: Intensive Lifestyle; M: Month; NA: Not Applicable; PCP: Primary Care Provider.
